# Perceived entrapment predicts first-onset suicidal ideation: A longitudinal study among medical students in China

**DOI:** 10.3389/fpubh.2022.1049975

**Published:** 2023-01-19

**Authors:** Suping Wang, Ting Wei, Rui Zhu, Sicong Li, Xinyi Liu, Yong Cai, Ruijie Gong

**Affiliations:** ^1^Tongji Hospital, School of Medicine, Tongji University, Shanghai, China; ^2^School of Medicine, Shanghai Jiao Tong University, Shanghai, China; ^3^Hongqiao International Institute of Medicine, Tongren Hospital, Shanghai Jiao Tong University School of Medicine, Shanghai, China; ^4^Center for Community Health Care, Hospital Development Institute Shanghai Jiao Tong University, Shanghai, China; ^5^Department Immunization Program, Xuhui Center for Disease Control and Prevention, Shanghai, China

**Keywords:** entrapment, suicidal ideation, medical students, prediction, longitudinal study

## Abstract

**Introduction:**

The prevalence of suicidal ideation among medical students is high. Evidence indicates that feelings of entrapment are a predictor of suicidal ideation. In this study, we aimed to (1) investigate the prevalence of first-onset suicidal ideation among Chinese medical students and (2) explore the predictive effects of perceived entrapment on first-onset suicidal ideation.

**Methods:**

This longitudinal study was conducted between 2018 and 2019 among 211 newly enrolled medical students in Shanghai. Using an anonymous questionnaire, we collected information on sociodemographic (sex, major, parents' income, and academic performance) and psychological (entrapment, depression, loneliness, defeat, social support, and interpersonal needs) variables as well as suicidal ideation. Participants were divided into four subgroups based on their exposure to entrapment (control, new-onset, reduced, and persistent). The primary outcome, first-onset suicidal ideation, was defined as suicidal ideation absent at baseline but present at follow-up.

**Results:**

In total, 54.98% of participants (116/211) were women, and 76.78% (162/211) majored in clinical medicine. In the follow-up survey, 6.16% of participants (16/211) reported first-onset suicidal ideation, 17.54% (37/211) reported new-onset entrapment, and 12.80% (27/211) reported persistent entrapment during follow-up. Compared with the control group who reported no perceived entrapment at baseline and follow-up, participants who reported new-onset entrapment had the highest risk of new-onset suicidal ideation [odds ratio (OR) = 14.700, 95% confidence interval (CI) = 2.906–74.364; adjusted OR = 8.798; 95% CI = 1.588–48.757; multivariate OR = 8.238, 95% CI = 1.394–48.693).

**Conclusion:**

New-onset entrapment can significantly predict suicidal ideation. Therefore, greater attention is needed for new-onset entrapment, such as intervention for suicidal ideation.

## 1. Introduction

Suicidal behavior (including suicidal ideation, suicide plans, suicide attempts, and suicides) has aroused widespread concern as a threat to global health. Given their length of medical training and relatively onerous academic burden (1), medical students have a higher risk of suicidal ideation, with a global prevalence of 11.1% according to a systematic review (2) and a prevalence of 17.9% in China (3), rates that are strongly predictive of suicide attempts and deaths (4). As pointed out by Blacker (5), suicide rates among medical students are infrequently reported in the historical and international literature, which indicates that the prevalence is far beyond what is reported. Consequently, exploring the generation and features of suicidal ideation among medical students is imperative to develop future intervention targets (6).

Suicidal ideation is a broad term defined as thinking about, considering, or planning suicide (7). Decades of studies showed that the development of suicide ideation is complex (4, 8). A wide range of psychological and social factors act together and lead to suicidal ideation (9), including loneliness (10), unmet interpersonal needs (11), depression (12), low self-esteem (13), and perceived defeat and entrapment (14).

The integrated motivational–volitional (IMV) model of suicidal behavior was proposed in 2011 and refined in 2018 to address this issue. Building upon the cry of pain (CoP) hypothesis (15), the motivational phase focuses on feelings of defeat and entrapment as key drivers of suicidal ideation. Defeat has been defined as a sense of failed social struggle and loss of or reduction in social rank (16). This may be directly related to interpersonal conflict but may also be related to perceptions of failure to attain social resources, including material resources (17). Feeling entrapped is a desire to escape from the current situation and the perception that all escape routes are blocked (16). This induces a strong motivation to escape from the feeling of being blocked (16) and comprises two components: external entrapment (the feeling of being blocked by external factors such as the living environment, others' opinions, or obligations) and internal entrapment (one's internal feelings and thoughts) (14, 18, 19). According to the IMV model, defeat and humiliation transit to entrapment with certain moderators, including poor coping and poor problem-solving (8); entrapment then translates to suicidal ideation with the enhancement or reduction role of a motivational moderator (20). Recent years have witnessed a burgeoning interest in entrapment and its potential mechanism for generating suicidal ideation. For example, in a cross-sectional study among Chinese adolescents, feelings of entrapment proved to be significantly related to suicidal ideation as were factors such as thwarted feelings of belongingness and perceived burdensomeness that could reinforce relationships (21).

Furthermore, it has been proven that entrapment mediates the relationship between defeat and suicidal ideation, with resilience as a possible alleviating factor (22, 23). Although many previous studies investigated the entrapment–suicidal ideation relationship, few focused on medical students, a population that is at higher risk of suicide (24). Additionally, most studies were cross-sectional (2, 20), focusing only on correlations of entrapment with suicidal ideation rather than longitudinal studies that investigate changes in the level of perceived entrapment in cases of suicidal ideation. A 4-month follow-up study revealed the role of entrapment in predicting suicidal ideation (25), which prompted us to probe this relationship over a longer period with participants categorized into groups based on levels of exposure to entrapment. Another disadvantage of previous studies is that few have been conducted in Asia or in low- and middle-income countries (26), which account for a large portion of global suicide deaths. Thus, the scope of longitudinal research on suicidal ideation must be broadened in terms of countries and regions. To the best of our knowledge, no prospective studies assessing entrapment level as a predictor of suicidal ideation among medical students have been conducted.

We designed a 12-month follow-up longitudinal study to (1) investigate the prevalence of first-onset suicidal ideation among Chinese medical students and (2) explore the predictive effect of perceived entrapment on first-onset suicidal ideation.

## 2. Materials and methods

### 2.1. Participants and procedures

The setting of the present study was a well-known medical school in Shanghai, China. All eligible freshmen who met the inclusion criteria were invited to participate in the baseline survey during their first year of university (*N* = 656). The progress of the investigation was reported in our previous study (27). The survey was distributed and collected online. One researcher worked with staff from the Student Work Service and trained survey administrators in the use of the questionnaire, on how to adequately explain the purpose and requirements of the survey, and on emphasizing the anonymous nature of the survey to allay respondents' concerns. The baseline (T0) survey was conducted from March to May 2018. Because the questionnaire was anonymous, the last four and three digits of participants' identity card numbers and phone numbers, respectively, were collected for matching purposes only. All eligible freshmen who met the inclusion criteria were invited to complete the follow-up survey (T1) during their second year of university (from June to July 2019).

**Figure 1:**
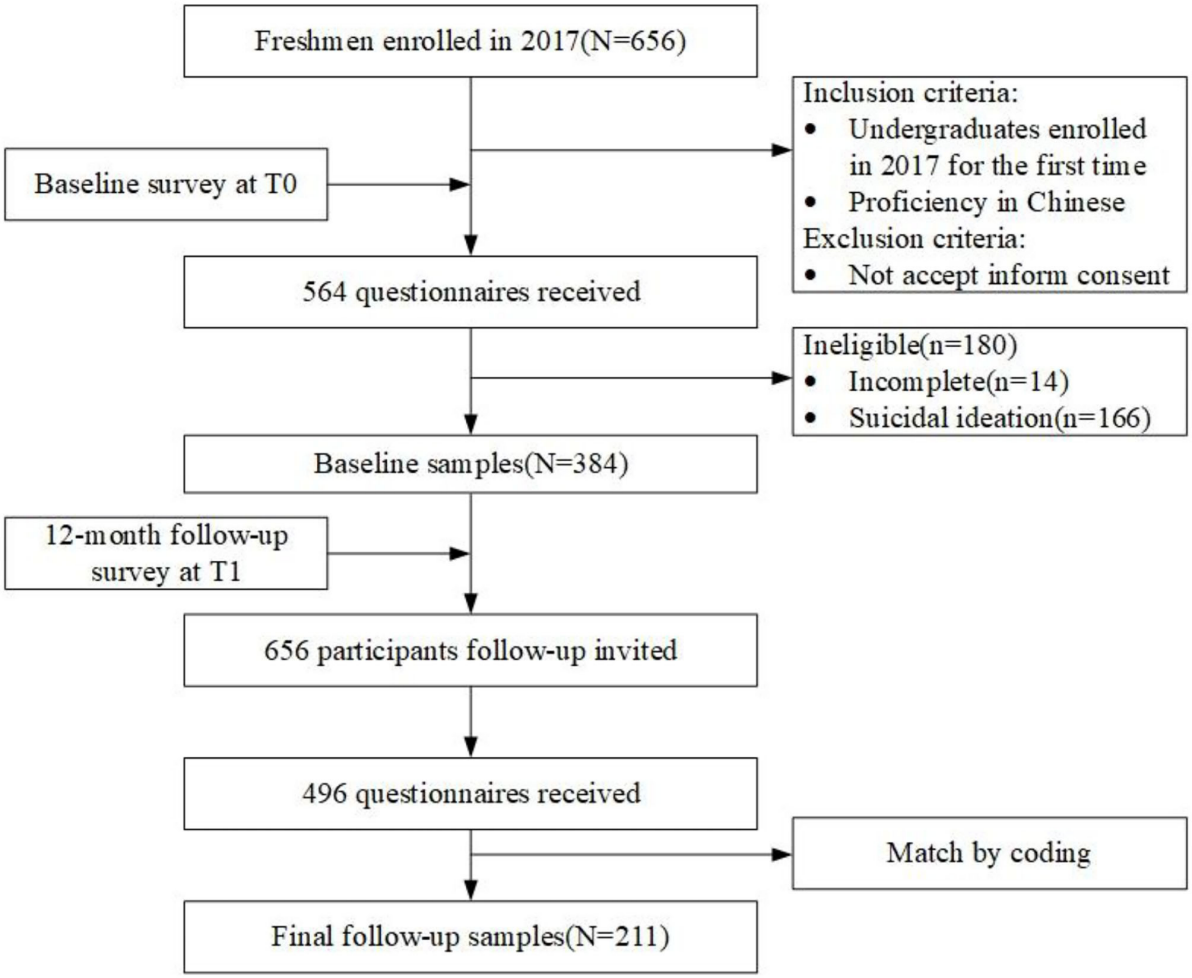
A flowchart of the study population. T0, baseline survey; T1, follow-up survey.

Questionnaires with any missing items were considered invalid. Casual samples were also excluded during data processing. Any participant who reported ever having suicidal ideation was excluded at baseline. Finally, the final 12-month follow-up cohort included 211 students (see Figure 1).

The inclusion criteria were as follows: (1) undergraduates enrolled in medical school for the first time during 2017 and (2) proficiency in Chinese.

### 2.2. Ethics

This study was approved by the Ethics Committee of the Shanghai Jiao Tong University School of Medicine (SJUPN-201813). Participants were guaranteed that the researchers would only access the collected data. Informed consent forms were signed by students who agreed to participate in this investigation. All participants were free to ask questions and withdraw if they did not wish to continue. Participants were also made aware that the last four and three digits of their identity card number and phone number, respectively, would be collected for matching purposes only and that they could not be identified with this information.

### 2.3. Measures

#### 2.3.1. Sociodemographic variables

Sociodemographic variables were measured at the baseline survey, including sex (male and female), major (clinical medicine and non-clinical medicine), parents' income (high, average, low), and academic performance (good: first third in class, average, or poor: last third in class).

#### 2.3.2. Psychological variables

##### 2.3.2.1. Entrapment

Entrapment was assessed using the Chinese version of the Entrapment Scale (16), a 16-item Likert-type questionnaire. The questionnaire focuses on the subjective feeling of entrapment, with each item ranging from 0 (not at all like me) to 4 (extremely like me). Questions 1–10 are focused on external entrapment, and questions 11–16 concentrate on internal entrapment. A higher total score indicates a higher level of perceived entrapment; the cut-off point was set to 16, and the upper quartile of entrapment scores was at T0. Participants with entrapment scores higher than 16 were defined as having perceived entrapment at the time of the investigation. The Chinese version of the Entrapment Scale has been proven valid and reliable among Chinese medical students (28) (Cronbach's alpha = 0.951; range 0–64).

##### 2.3.2.2. Depression

The level of depression was measured using the Chinese version of the 9-item Patient Health Questionnaire (PHQ-9) (29). Participants were asked about the frequency of depressive symptoms in the past 2 weeks. Each item's score ranged from 1 (not at all) to 4 (almost every day) on a Likert-type scale. As the total score increased, the participant's degree of depression became more severe. With reference to previous research (29), scores above 13 were considered to indicate depression. The Chinese version of the PHQ-9 questionnaire showed good reliability and validity among college students (30) (Cronbach's alpha = 0.871; range 9–36).

#### 2.3.2.3.Loneliness

The 8-item UCLA Loneliness Scale (ULS-8) was used to evaluate loneliness among participants (31). Items are rated using a Likert scale, with scores ranging from 1 (not at all) to 4 (almost every day). The items concentrate on participants' subjective feelings of loneliness. A higher total score corresponds to a higher level of loneliness. Because no standard cutoff point is available, the upper quartile score (T0) of 17 was set as the cutoff. Participants with ULS-8 scores higher than 17 were defined as having a relatively high level of perceived loneliness at the time of the investigation (Cronbach's alpha=0.829; range 8–29).

##### 2.3.2.4. Defeat

The Defeat Scale was used to assess participants' feelings of defeat (16). The questionnaire comprises 16 items, with each item ranging from a score of 0 (never) to 4 (always). The Chinese version of the Defeat Scale has been proven valid and reliable among Chinese medical students (32). A higher total score indicates more despondent feelings of defeat. The upper quartile (T0) score of 18 was used as the cutoff point. Participants with defeat scores higher than 18 were defined as having relatively high levels of perceived defeat at the time of investigation (Cronbach's alpha=0.907; range 0–52).

##### 2.3.2.4.Social support

The Multidimensional Scale of Perceived Social Support (MSPSS) was used to measure participants' social support. The MSPSS comprises 12 questions regarding participants' subjective feelings of support from family, friends, and others. The MSPSS has been widely used in multiple countries with good reliability and validity (33, 34). A higher total score indicates greater social support; hence, the lower quartile score (T0), 67, was chosen as the cutoff point. Participants with MSPSS scores lower than 67 were defined as having relatively low levels of perceived social support at the time of investigation (Cronbach's alpha=0.960; range 33–84).

##### 2.3.2.5. Interpersonal needs

The 15-item Interpersonal Needs Questionnaire (INQ-15) includes six questions to measure perceived burdensomeness and nine questions to assess thwarted belongingness (35); scores range from 1 (strongly disagree) to 7 (strongly agree). With six items reverse-scored, a higher total score indicates greater thwarted belongingness and perceived burdensomeness in the respondent. The cutoff was set at an upper quartile score (T0) of 37. Participants with the INQ-15 scores higher than 37 were defined as having a relatively high level of unmet interpersonal needs at the time of the investigation. We used the Chinese version of the questionnaire, which showed good reliability and validity among Chinese college students (36) (Cronbach's alpha=0.895; range 15–78).

#### 2.3.3. Outcome

##### 2.3.3.1. First-onset suicidal ideation

Suicidal ideation was measured by asking students: “Have you ever had thoughts of taking your own life?” (T0) and “During the past 12 months, have you had thoughts of taking your own life?” (T1) (37). A “yes” response to the first question was taken to indicate the existence of lifetime suicidal ideation. A response of “yes” to the latter question was considered to indicate suicidal ideation during the previous 12 months. Thus, first-onset suicidal ideation was defined as suicidal ideation absent at T0 but present at T1.

### 2.5. Statistical analysis

To estimate the association between entrapment and first-onset suicidal ideation, the 211 participants were divided into four subgroups according to their levels of entrapment. All covariates except entrapment were collected simultaneously when the outcome was measured (T0). We used IBM SPSS version 26.0 for Windows (IBM Corp., Armonk, NY, USA) in descriptive analysis and logistic regression. Several logistic regression models were also conducted to examine differences among subgroups. Statistical significance was set at a *p*-value of < 0.05.

## 3. Results

### 3.1. Sociodemographic and psychological characteristics of participants

The mean participant age ± standard deviation was 19.75 ± 0.673 years. As shown in Table 1, 54.98% of participants (116/211) were women, and 76.78% (162/211) majored in clinical medicine. Most students (65.88%,139/211) considered their parents' income to be average, and approximately half (58.29%,123/211) reported average academic performance. The rates of perceived entrapment, depression, loneliness, defeat, social support, and unmet interpersonal needs were 21.80% (46/211), 45.02% (95/211), 23.70% (50/211), 24.64% (52/211), 73.93% (156/211), and 24.17% (51/211), respectively.

**Table 1:** Sociodemographic and psychological variables associated with suicidal ideation.

		**Frequency**	**Percentage**	**OR (95% CI)**
**Sociodemographic variables**				
Gender				
	Men	95	45.02	0.346 (0.092–1.294)
	Women	116	54.98	Ref.
Major				
	Clinical medicine	162	76.78	0.662 (0.195–2.250)
	Non–clinical medicine	49	23.22	Ref.
Parents' income			
	Low	26	12.32	0.573 (0.057–5.812)
	Average	139	65.88	0.992 (0.257–3.833)
	High	46	21.80	Ref.
Academic performance			
	Poor	35	16.59	0.900 (0.201–4.032)
	Average	123	58.29	0.407 (0.113–1.469)
	Good	53	25.12	Ref.
**Psychological variables**				
Entrapment			
	High	46	21.80	1.651 (0.484–5.626)
	Low	165	78.20	Ref.
Depression			
	High	95	45.02	4.431 (1.183–16.597)^*^
	Low	116	54.98	Ref.
Loneliness			
	High	50	23.70	5.943 (1.848–19.112)^**^
	Low	161	76.30	Ref.
Defeat			
	High	52	24.64	2.008 (0.627–6.433)
	Low	159	75.36	Ref.
Social support			
	Low	55	26.07	2.606 (0.836–8.127)
	High	156	73.93	Ref.
Interpersonal needs			
	High	51	24.17	2.914 (0.932–9.112)
	Low	160	75.83	Ref.
First-onset suicidal ideation				
	Yes	13	6.16	
	No	198	93.84	

^*^p < 0.050;

^**^p < 0.010. OR, odds ratio; CI, confidence interval; Ref., reference category.

In total, 6.16% of participants (16/211) reported having suicidal ideation in the past year. Among all variables measured at baseline, only depression and loneliness showed a significant relationship with first-onset suicidal ideation [odds ratio (OR) = 4.431 and 5.943, respectively).

### 3.2. Suicidal ideation among subgroups

Table 2 shows that 6.16% of the total participants (13/211) reported suicidal ideation in the past year. We found significant differences in the prevalence of suicidal ideation among the four subgroups (χ^2^ = 16.269, *p* = 0.001), and participants with new-onset perceived entrapment at T1 had the highest prevalence (18.92%, 7/37). There were no significant differences in sociodemographic characteristics among the four subgroups, except for sex.

**Table 2:** Subgroup division and suicidal ideation in the four subgroups.

**Group**	**Entrapment status**		**Group size**	**Suicidal ideation**	
	**T0**	**T1**		**Number**	**Raw%**
Control	–	–	128	2	1.56%
Reduced	+	–	19	1	5.26%
New-onset	–	+	37	7	18.92%
Persistent	+	+	27	3	11.11%
Total			211	13	6.16%

(–) No perceived entrapment; (+) perceived entrapment; T0, baseline survey; T1, follow-up survey.

### 3.3. Logistic regression models for suicidal ideation

The results of logistic regression models for entrapment and suicidal ideation are presented in Table 3. Compared with the control group who reported no perceived entrapment at T0 and T1, participants who reported new-onset entrapment at T1 had the highest risk of new-onset suicidal ideation [OR of model 1 = 14.700, 95% confidence interval (CI) = 2.906–74.364; adjusted OR = 8.798, 95% CI = 1.588–48.757; multivariate OR = 8.238, 95% CI = 1.394–48.693). Participants who perceived persistent entrapment at T0 and T1 also had a higher risk of developing suicidal ideation (OR = 7.875, 95% CI = 1.555–27.175 without any adjustment).

**Table 3:** Models of entrapment exposure with risk of suicidal ideation.

**Group**	**Model 1**			**Model 2**			**Model 3**		
	**p**	**OR** ^a^	**95% CI**	**p**	**ORa** ^b^	**95% CI**	**p**	**ORm** ^c^	**95% CI**
New–onset	0.001	14.700	2.906–74.364	0.013	8.798	1.588–48.757	0.020	8.238	1.394–48.693
Reduced	0.316	3.500	0.302–40.589	0.694	1.677	0.128–21.947	0.736	1.669	0.085–32.906
Persistent	0.028	7.875	1.249–49.668	0.282	3.037	0.401–23.014	0.388	3.017	0.246–37.034
Control	–	Ref.	–	–	Ref.	–	–	Ref.	–

^a^OR, odds ratio of Model 1, including only entrapment.

^b^ORa, the adjusted odds ratio of Model 2, including entrapment and significant variables (depression and loneliness), measured at T0.

^c^ORm, the multivariate odds ratio of Model 3, including entrapment and all other variables measured at T0 (sex, age, parents' income, academic performance, defeat, loneliness, depression, interpersonal needs, and social support).

## 4. Discussion

The prevalence of first-onset suicidal ideation among medical students in their second year of school was reported at a rate of 6.16% in our study, lower than the crude summary prevalence of 11.1% among medical students during medical school (2). Our study findings revealed that new-onset perceived entrapment is a robust predictor of first-onset suicidal ideation. In line with our results, multiple studies and models have demonstrated the importance of entrapment in the development of suicidal ideation (22, 38); however, past studies varied with respect to participants and the psychological variables investigated (20, 25, 39, 40). Most previous research involved cross-sectional studies, although several longitudinal studies have been conducted for real-time observation of variability in certain psychological factors by assessing participants multiple times per day (25, 41, 42). One such survey investigated 1,239 high school students in Guangxi and Guangdong provinces of China (21) and showed that entrapment was significantly related to suicidal ideation. A study among participants aged 18–80 years in Germany (43) and another among university students in Spain (44) demonstrated that entrapment could predict suicidal ideation. Limited long-term longitudinal research on this topic has been carried out in China; among these, our study is the first with a follow-up period of up to 1 year. In comparison with existing research, a novel approach in the present longitudinal survey was to divide the level of exposure to entrapment according to four classifications to explore the relevance between different stages of entrapment and suicidal ideation. Our research demonstrated the close relationship between entrapment, especially new-onset entrapment, and first-onset suicidal ideation, which supports the IMV model (45, 46), corroborating that perceived entrapment is an important predictor of first-onset suicidal ideation.

In our study, new-onset entrapment was mostly related to suicidal ideation, possibly because study participants who perceived new-onset entrapment had recently been feeling external or internal pressure, and they may have had strong subjective feelings of having no way to escape their situation and desperation. Participants who reported persistent entrapment may have been more inclined to become accustomed to a difficult situation, which may lower their likelihood of considering extreme measures, such as suicide. In accordance with the IMV model, protective motivational moderators may not yet have taken shape compared to the perception of persistent entrapment, which may have led to developing tolerance toward feeling entrapped. However, there is no valid basis for this assumption yet; further studies are needed to verify the association between new-onset entrapment and persistent entrapment with suicidal ideation.

Our current study had several limitations. First, as a longitudinal study with a follow-up period of 12 months, loss to follow-up was a problem. Only 32.16% (211/656) of eligible freshmen completed the follow-up survey. Second, the sample was restricted to freshmen at one medical school, leading to a relatively small sample size, which may contribute to sampling error. However, the small sample size guaranteed a more reliable follow-up. Third, the evaluation tool for suicidal ideation used in this study comprised a set of simplified questions from previous studies. The use of scales to evaluate not only suicidal ideation but also suicide behaviors may provide more in-depth findings. Although it is important to include as many factors as possible, a long questionnaire will affect its reliability. Therefore, we only included the most important factors in this study. Ultimately, the study results showed that entrapment may serve as a mediator while developing suicidal ideation.

Further research targeting this pathway is required to improve our understanding of the process of generating suicidal ideation and its intervention. Finally, although the assessment of mental disorders was based on self-reports, evidence shows good diagnostic agreement with clinical judgment (47). We suggest a clinical diagnosis for cases of “probable” mental disorders and further treatment (44).

## 5. Conclusion

Feelings of entrapment have been shown to be a risk factor for suicidal ideation, but these feelings tend to fluctuate. Therefore, regular follow-up to assess levels of perceived entrapment could be an effective way to screen for and prevent the development of suicidal ideation. With reference to the IMV model, individuals with perceived entrapment, especially new-onset entrapment, require special attention with respect to motivational moderators such as thwarted belongingness, burdensomeness, lack of future thoughts, lack of social support, and negative attitudes. Greater attention is needed for new-onset entrapment, such as with intervention for suicidal ideation.

## Data availability statement

The original contributions presented in the study are included in the article/supplementary material, further inquiries can be directed to the corresponding authors.

## Ethics statement

The study was approved by the Ethics Committee of Shanghai Jiao Tong University School of Medicine (SJUPN-201813). Informed, written consent was obtained from all individual participants included in the study. All methods were carried out in accordance with relevant guidelines and regulations.

## Author contributions

YC contributed to the conceptualization. SW contributed to the design of the work and supervision. TW and RZ contributed to the acquisition, analysis, and interpretation of data and the drafting of the manuscript. SL and XL contributed to data acquisition, analysis, and interpretation. RG contributed to the critical revision of the analysis and the drafting and editing of the manuscript. All authors contributed to the critical revision of the paper and approved the final manuscript for publication.
